# Hesperetin Prevents Bone Resorption by Inhibiting RANKL-Induced Osteoclastogenesis and Jnk Mediated Irf-3/c-Jun Activation

**DOI:** 10.3389/fphar.2018.01028

**Published:** 2018-09-11

**Authors:** Qiang Zhang, Xinqiao Tang, Zhong Liu, Xiaoxia Song, Dan Peng, Wei Zhu, Zhengxiao Ouyang, Wanchun Wang

**Affiliations:** ^1^Department of Orthopedics, The Second Xiangya Hospital, Central South University, Changsha, China; ^2^Department of Orthopedics, Xiangtan Central Hospital, Xiangtan, China; ^3^Department of Respiratory Medicine, Xiangtan Central Hospital, Xiangtan, China

**Keywords:** osteoporosis, hesperetin, RANKL, Irf-3, MAPK, NFATc-1

## Abstract

Bone homeostasis and resorption is regulated by the proper activation of osteoclasts, whose stimulation largely depends on the receptor activator of nuclear factor κB ligand (RANKL)-RANK signaling. Herein, for the first time, we showed that interferon regulatory factor (Irf)-3 was intimately involved in RANKL-induced osteoclast formation. In addition, hesperetin (Hes) derived from citrus fruit could inhibit RANKL-induced osteoclast differentiation and maturation among three types of osteoclast precursors with inhibited formation of F-actin rings and resorption pits on bone slices. More importantly, by using SP600125, a selective Jnk inhibitor, we showed that Hes was able to significantly attenuate the Jnk downstream expression of Irf-3 and c-Jun, thereby inactivating NF-κB/MAPK signaling and transcriptional factor NFATc-1, leading to suppression of osteoclast-specific genes, which resulted in impaired osteoclastogenesis and functionality. An ovariectomized (OVX) osteoporosis mouse model demonstrated that Hes could increase trabecular bone volume fractions (BV/TV), trabecular thickness, and trabecular number, whereas it decreased trabecular separation in OVX mice with well-preserved trabecular bone architecture and decreased levels of TRAP-positive osteoclasts. This is further evidenced by the diminished serum expression of bone resorption marker CTX and enhanced production of osteoblastic ALP *in vivo*. Taken together, these results suggested that Hes could inhibit Jnk-mediated Irf-3/c-Jun activation, thus attenuating RANKL-induced osteoclast formation and function both *in vitro* and *in vivo*.

## Introduction

Bone is a dynamic tissue that undergoes a series of anabolic and metabolic steps to harbor bone marrow, store minerals, and protect inner organs (Hu et al., 2018). Bone homeostasis is orchestrated by the active balance between osteoblast formation and osteoclast resorption, which contributes to normal skeletal physiology and functionality. However, certain disturbing stimuli within the bone microenvironment such as tumors, endocrine disorders, osteoarthritis and aging (Liu et al., 2017; Qiao et al., 2017; Qu et al., 2018) inevitably result in disruption in the equilibrium between osteoblasts and osteoclasts, leading to various skeletal disease phenotypes such as osteopenia, osteoporosis, and osteopetrosis (Sobacchi et al., 2013; Vegger et al., 2018), which significantly affect the life-quality and life-span of patients.

Derived from the monocyte-macrophage hematopoietic linage, osteoclast precursors are stimulated by the receptor activator of nuclear factor κB ligand (RANKL) and macrophage colony-stimulating factor (MCSF) for further activation, maturation and differentiation (Zhao et al., 2007; Xia et al., 2011). It has been demonstrated that MCSF is responsible for osteoclast survival, and also supports the increased expression of RANK on osteoclast cell membrane surfaces for RANKL binding (Xing et al., 2012), which, together, are intimately correlated with osteoclastogenesis. Upon binding RANKL to RANK, TNF receptor-associated factor 2/6 (TRAF 2/6) and transforming growth factor β-activated kinase 1 (TAK1) are activated for subsequent phosphorylation of IκB kinase (IKK) complexes, c-Jun N-terminal kinase (Jnk), extracellular-signal regulated kinase (Erk) and p38, activating NF-κB and mitogen-activated protein kinase (MAPK) signaling to facilitate osteoclastogenesis (Boyle et al., 2003; Feng, 2005). Nonetheless, in spite of the fact that much research performed to date has unraveled the mechanisms of action during osteoclast formation in detail, there is still an urgent need to screen novel molecular targets for comprehensively understanding the process of osteoclast formation, and for developing future customized therapies. Herein, interferon regulatory factor (Irf)-3 emerges as a key molecule in regulating innate immune responses and has been found to be associated with the expression of NF-κB and MAPK(Zhang et al., 2009). It was shown that by targeting TANK-binding kinase (TBK-1) and IKK complex, measles virus nucleocapsid protein (MVNP) could activate both Irf-3 and NF-κB to increase osteoclast numbers (Sun et al., 2014). However, no studies have been conducted to further validate the direct significance of Irf-3 during RANKL-induced osteoclastogenesis. Hence, an in-depth insight of Irf-3 during osteoclastogenesis is urgently required, which would undoubtedly provide novel and effective targets for future osteopenia research and clinical practice.

Hesperetin (Hes), a glycoside flavonoid from the flavanone family that is derived from citrus fruit (Silveira et al., 2014), has been demonstrated to exert immune-regulatory (MacGregor et al., 1983), anti-tumor (Coutinho et al., 2017), and anti-inflammatory (Comalada et al., 2006) effects. Importantly, several studies showed that Hes was capable of promoting osteogenic differentiation of both human and rat mesenchymal stem cells via Erk/Smad/BMP pathways (Trzeciakiewicz et al., 2010; Xue et al., 2017). However, besides the promising osteoblastogenic effect of Hes, little is known about the inhibitory effects of Hes against RANKL-induced osteoclastogenesis and especially the underlying mechanisms involved. Here, aiming at providing not only novel molecular targets for future osteoporosis treatment, but also effective pharmaceutical remedies to inhibit osteoclastogenesis, we previously screened several drugs and found that Hes could attenuate RANKL-induced osteoclastogenesis by regulating Irf-3 mediated activation of Jnk, c-Jun and NFATc-1 in osteoclasts, thereby extending the spectrum of translational osteoporosis treatment on both the pharmaceutical and molecular levels.

## Materials and Methods

### Main Reagents and Cell Culture

Hesperetin (Hes) was purchased from Dalian Meilun Biotechnology (Liaoning, China), dissolved in dimethyl sulfoxide (DMSO), and kept from direct light. RANKL and recombinant MCSF were purchased from R&D Systems (Minneapolis, MN, United States). The selective inhibitor of Jnk, SP600125, was obtained from Sigma-Aldrich (St. Louis, MO, United States). Primary and secondary antibodies for western blotting and immunohistochemistry were purchased from Cell Signaling Technology (Cambridge, MA, United States). CTX Elisa Kit was purchased from Novus Biologicals (Littleton, CO, United States). ALP Elisa Kit was purchased from Abcam (Cambridge, United Kingdom).

MC3T3-E1 and osteoclast precursor RAW 264.7 cells that obtained from American Type Culture Collection (ATCC) were cultured in α-MEM medium supplemented with 10% FBS (Gibco, Invitrogen Ltd., Carlsbad, CA, United States) and 1% penicillin/streptomycin. C57BL/6/Bkl mice (female, 8 weeks old) of pure genetic background that obtained from Shanghai Laboratory Animal Company, CAS (SLACCAS, Shanghai, China) were used to harvest (1) primary bone marrow monocytes (BMMs) from the tibias and femurs and (2) primary splenocytes from thoroughly ground spleen tissue to induce osteoclastogenesis *in vitro*. Both primary BMMs and splenocytes were cultured in α-MEM medium supplemented with 30 ng/ml MCSF and 10% FBS. All cells were passaged at approximately 80% confluence and incubated at 37°C in a high-humidity atmosphere of 5% CO_2_.

### Cell Viability

We used the Cell Counting Kit-8 (CCK-8) method (Zhou et al., 2018) to assess viability of osteoclastic RAW 264.7 cells after Hes treatments. RAW 264.7 cells were seeded into 96-well plates at 6000 cells/well overnight. Next, various concentrations of Hes were added (200, 100, 50, 25, 12.5, 6.25, 3.125, 1.5, and 0.75 μM) into wells. After 24 h, 10 μL CCK-8 solution (KeyGen Biotech, Nanjing, China) was added according to the manufacturer’s protocol. Absorbance at 450 nm was measured via a microplate reader (Thermo Electron Corp, Waltham, MA, United States).

### *In vitro* Osteoclastogenesis Assessment

Here, we used three osteoclast precursor cell types to fully evaluate the osteoclastogenesis inhibitory effects of Hes after RANKL stimulation. RAW 264.7 cells, splenocytes, and BMMs were seeded in 96-well plates and treated with Hes at varying non-toxic concentrations from 15 to 60 μM. RANKL (50 ng/ml) was used to stimulate preosteoclasts for up to 10 days. After osteoclast differentiation, cells were fixed in 4% paraformaldehyde (PFA) and labeled by tartrate-resistant acid phosphatase (TRAP) staining. Stained osteoclasts with at least three nuclei were classified as being TRAP-positive, indicating matured and differentiated multi-nucleated osteoclasts.

### F-actin Ring Formation

F-actin ring immunofluorescence was evaluated to illustrate the ruffled membrane of osteoclasts (Xiao et al., 2015). BMMs were induced under stimulation of 50 ng/ml RANKL and varying concentrations of Hes (15, 30, and 60 μM). After differentiation, osteoclasts were fixed in 4% PFA and permeabilized in 0.1% Triton X-100. The cytoskeletons of osteoclasts were indicated by Alexa Fluor 647 phalloidin staining and osteoclast cell nuclei were marked by DAPI. Following extensive washing with PBS, confocal microscopy (Leica TCS SP8, Germany) was then used to investigate the fluorescent formation of F-actin rings.

### Bone Resorption Pit Evaluation

BMMs (8000 cells/slice) were planted onto bovine bone slices and treated with Hes at varying dosages from 15 to 60 μM. In addition, 50 ng/ml RANKL and 50 ng/ml MCSF were administered to BMMs for osteoclast formation. After positive labeling of TRAP^+^ osteoclasts in a control group, adherent osteoclasts were completely removed by sonication and mechanical agitation. The osteolytic bone resorption pits were observed under scanning electron microscopy (SEM).

### Alkaline Phosphatase (ALP) Activity and Staining

For osteoblastic differentiation, MC3T3-E1 cells were treated with osteogenic medium of 10 mM β-glycerophosphate, 0.1 μM dexamethasone and 50 μg/mL ascorbic acid (Sigma-Aldrich, United States) according to previous reports (Pan et al., 2018). The medium was added with Hes (low: 15 μM; high: 30 μM) and changed every 2 days throughout the study. After 14 days of incubation, osteoblasts were rinsed with sterile PBS completely, followed by the treatment of 0.2% Triton-X100 solution for 4 standard freeze-thaw cycles. The ALP activity in lysis solution was then determined by colorimetric assay based on p-nitrophenyl phosphate. A MicroBCA protein assay kit (Pierce Biotechnology, Thermo Fisher Scientific, Waltham, MA, United States) was deployed to investigate and normalize the intracellular protein level.

We used an ALP staining kit (Renbao, Shanghai, China) at 14th day to stain osteoblasts. Cells were fixed with 4% PFA and washed with PBS for several times. Next, staining reagents were used to mark osteoblastic cells according to manufacture’s protocols. Afterward, specimens were rinsed completely for visualization under microscope.

### Quantitative Real-Time PCR

To investigate osteoclast-specific gene expression, BMMs were seeded in 6-well plates and treated with Hes at 15 μM (Low) and 30 μM (High) for subsequent extraction of total RNA via RNeasy Mini Kit (Qiagen, Valencia, CA, United States) following the manufacturer’s guidance. A reverse transcriptase kit (Takara Biotechnology, Japan) was used to synthesize cDNA. SYBR Premix Ex Taq Kit (Takara Biotechnology) was employed to conduct qPCR using an ABI 7500 Sequencing Detection System (Applied Biosystems, Foster City, CA, United States) according to instrument instructions. PCR was conducted under following conditions: 5 s of denaturation at 95°C, 34 s of amplification at 60°C, 40 cycles. The data was analyzed with delta-delta CT method. The murine osteoclastic primers used were listed in **Table 1**.

**Table 1:** Primer sequences for real-time PCR analysis.

Gene	Forward primer (5′-3′)	Reverse primer (5′-3′)	Product Length/bp
*Dcstamp*	AAAACCCTTGGGCTGTTCTT	AATCATGGACGACTCCTTGG	190
*Calcr*	CGGACTTTGACACAGCAGAA	AGCAGCAATCGACAAGGAGT	188
*Nfatc-1*	CCGTTGCTTCCAGAAAATAACA	TGTGGGATGTGAACTCGGAA	152
*Traf-6*	AAACCACGAAGAGGTCATGG	GCGGGTAGAGACTTCACAGC	175
*c-Fos*	CCAGTCAAGAGCATCAGCAA	AAGTAGTGCAGCCCGGAGTA	247
*Acp5*	TCCTGGCTCAAAAAGCAGTT	ACATAGCCCACACCGTTCTC	212
*Ctsk*	CTTCCAATACGTGCAGCAGA	TCTTCAGGGCTTTCTCGTTC	155
*Atp6v0a3*	GCCTCAGGGGAAGGCCAGATCG	GGCCACCTCTTCACTCCGGAA	120
*Atp6v0a2*	TTCCTTGGAGCCCCTGAGCACAT	TGTGAAACGGCCCAGTGGGTG	145
*Gapdh*	ACCCAGAAGACTGTGGATGG	CACATTGGGGGTAGGAACAC	171

### Western Blotting

To explore the role of Irf-3 during osteoclastogenesis, we seeded RAW264.7 cells in 6-well plates at a density of 5 × 10^5^ cells/well. For analyzing NF-κB/MAPK activation, RAW264.7 cells were treated with 50 ng/ml RANKL, 30 μM Hes alone and in combination for 30 min. DMSO-treated osteoclasts were then used as controls. To determine the crosstalk between Irf-3 and NFATc-1, c-Jun and c-Fos, RAW264.7 cells were treated with 50 ng/ml RANKL with or without 30 μM Hes for 0, 1, and 3 days. In addition, SP600125, a selective Jnk inhibitor, was utilized at non-toxic dosages to evaluate the effect of Hes in Jnk/Irf-3/c-jun-mediated NFATc-1 activation. Untreated osteoclasts were also used as controls.

We used radioimmunoprecipitation assay (RIPA) lysis buffer (Beyotime Bioscience, Shanghai, China) with added phenylmethysulfonyl fluoride (PMSF) to collect osteoclast total protein. The extracted lysates were quantified using a BCA Assay Kit (Thermo Scientific, Waltham, IL, United States) and denatured at 99°C for 10 min. Then, 30 μg of proteins were resolved by sodium dodecyl sulfate polyacrylamide gel electrophoresis (SDS-PAGE) on 10–12.5% precast gels at 80 and 120 V for 0.5 and 1 h respectively. Next, gels were transferred to a methanol-activated polyvinylidene fluoride (PVDF) membrane for 2.5 h at constant 250 mA. Membranes were then blocked by Tris-buffered saline with 0.05% Tween (TBST) and 5% non-fat milk powder for 2 h. After the sequential incubation of membranes with primary antibody at 4°C overnight and relevant secondary antibody for 2 h, TBST was repeatedly used to rinse membranes. In the absence of direct light stimulation, the Odyssey Infrared Imaging System (LI-COR Bioscience, Lincoln, NE, United States) was employed to visualize the expression of specific osteoclast proteins after Hes treatments.

### NF-κB Luciferase Activity

RAW264.7 cells transfected with NF-κB luciferase constructs were administered with 50 ng/ml RANKL and 30 μM Hes for 3 days. NF-κB luciferase activity was measured using a Promega Luciferase Assay System (Madison, WI, United States) following the manufacturer’s guidance.

### Ovariectomized (OVX) Murine Osteoporosis Model

This study was carried out in accordance with the recommendations of guiding principles of Animal Care Committee of Central South University and protocol was also approved by the Animal Care Committee of Central South University. A total of 40 female C57BL/6 mice (12 weeks old) obtained from Shanghai Laboratory Animal Company, CAS (SLACCAS, Shanghai, China) were nurtured in specific pathogen-free (SPF) plastic-isolator cages before ovarian resection. Prior to surgery, mice were kept under anesthetization by ketamine (80 mg/kg.bw, Hengrui Medicine Co Ltd., Jiangsu, China) and xylazine (10 mg/kg.bw, Baide Biomedicine, Qingdao, China) administered intraperitoneally, followed by a retroperitoneal incision that was ventral to the erector spinae caudal to the last rib. Such procedures offered ready access to the ovaries, which enabled their removal with catgut ligature around the cranial portions of the uterus and uterine vessels. After surgery, the incision was closed and OVX mice were kept in warmed cages for recovery.

Ovariectomized mice were randomly divided into four groups: Ctrl group (only with PBS injection), Vehicle group (osteoporosis mice with PBS injection), Low group [osteoporosis mice with 6 mg/kg bodyweight (bw), Hes injection intraperitoneally, 3 times/week] and High group [osteoporosis mice with 12 mg/kg.bw, Hes injection intraperitoneally, 3 times/week] (*n* = 10/group). All animals were observed for 8 weeks and sacrificed at the end. Right tibias were harvested for subsequent μCT scanning.

### μCT Scanning

After sacrifice of animals, the collected right tibias were fixed in 4% PFA for 48 h and processed for high-resolution μCT analyses (μCT 40, Scanco, Zurich, Switzerland) with the following parameters: 80 kV (X-ray voltage) and 80 μA (electric current) at 10 μm resolution. μCT analysis was blinded to the treatment group allocation. Relevant trabecular bone volume fractions (BV/TV, %), trabecular separation (Tb.Sp, mm), trabecular thickness (Tb.Th, mm), and trabecular number (Tb.N, 1/mm) were determined to investigate the *in vivo* effects of Hes on osteoporotic mice according to the ASBMR guidelines for assessment of bone microstructure in rodents using micro-computed tomography (Bouxsein et al., 2010).

### Immunohistochemical Staining Analyses

Fixed tibias were decalcified in 10% EDTA for 3 weeks and subsequently embedded in paraffin. Samples underwent serial sectioning and were stained with hematoxylin-eosin (H&E) and TRAP to indicate histomorphometric changes after Hes administration *in vivo*.

### Enzyme-Linked Immunosorbent Assay (ELISA)

Serum was collected from cardiac puncture of mice prior to the high-speed centrifuge at 12000 rpm for 15 min. The *in vivo* serum levels of bone resorption marker C-terminal telopeptide of type I collagen (CTX) and osteoblastic alkaline phosphatase (ALP) were measured via ELISA kits, according to the manufacturer’s instructions.

### Statistical Analyses

All data obtained were analyzed by SPSS 13.0 software (SPSS Inc., United States) using one-way analysis of variance (ANOVA) followed by Dunnett *post hoc* test to conduct comparisons between different groups. All experiments were repeated at least three times for biological replicates. Data are presented as means ± standard deviation (SD). A *p*-value ≤ 0.05 for two-tailed test was considered statistically significant.

## Results

### Suppression Against Osteoclast Formation and Function by Non-toxic Hes *in vitro*

**FIGURE 1:**
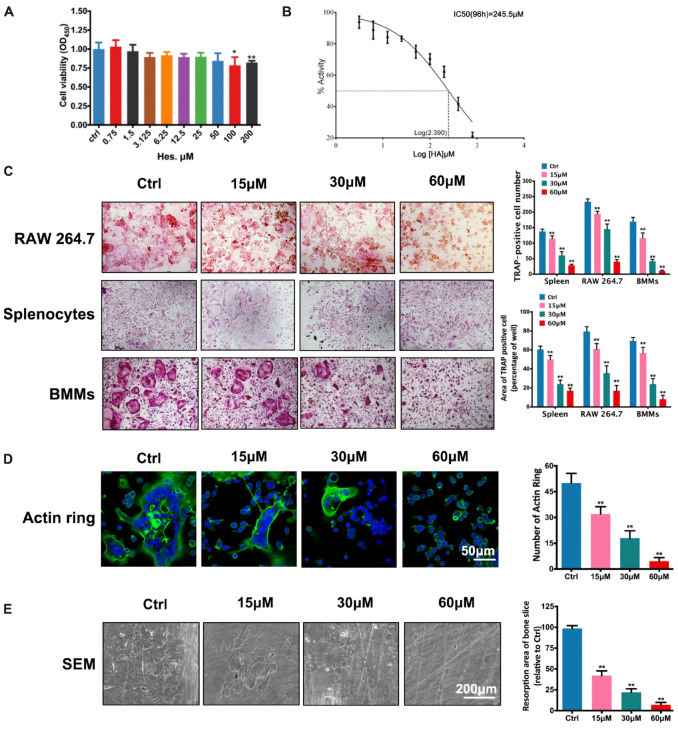
Non-toxic Hes attenuated RANKL-induced osteoclast formation and function *in vitro*. **(A)** Cell viability of osteoclast precursors after Hes treatments for 24 h. **(B)** Linear correlation between OD values and cell numbers. **(C)** RANKL-induced osteoclastogenesis after Hes treatments in three types of preosteoclasts, RAW 264.7 cells, splenocytes, and BMMs. **(D)** Formation of RANKL-induced F-actin rings after Hes treatments. **(E)** Formation of RANKL-stimulated bone resorption pits after Hes treatments. ^∗^*p* < 0.05 compared with controls, ^∗∗^*p* < 0.01 compared with controls.

In order to exclude the possibility of inhibited osteoclastogenesis caused by the decreased number of osteoclasts, we explored non-toxic concentrations of Hes against preosteoclasts for observing its anti-osteoclastogenesis effect. As shown in **Figure 1A**, we found that only high concentrations of Hes (100 and 200 μM) were capable of significantly decreasing osteoclast survival. Conversely, Hes of less than 50 μM failed to attenuate osteoclast proliferation compared with controls, indicating the potential range for selection in further osteoclast formation investigations. The IC_50_ of Hes toward RAW 264.7 cells was 245.5 μM at 96 h post-administration. Additionally, an excellent linear relationship (*R*^2^ = 0.9863) between OD values at 450 nm and cell numbers was found (**Figure 1B**), indicating the decreased values of OD_450_ could effectively reflect the inhibited cell proliferation by Hes treatments. Hence, Hes of 15, 30, and 60 μM were used for our remaining studies.

Next, inhibitory RANKL-induced osteoclastogenesis in various osteoclast precursors (RAW 264.7, BMMs, and splenocytes) by Hes treatment was investigated. Results showed that despite the formation of large, round, and red-stained multinucleated osteoclasts in control groups after RANKL stimulation, the addition of Hes markedly impeded the maturation of multinucleated osteoclasts in a dose-dependent manner, as exemplified by the decreased number and area of TRAP-positive osteoclasts for all three osteoclast types (**Figure 1C**), implicating that non-toxic Hes was able to significantly suppress osteoclast formation. It is acknowledged that normal osteoclast function requires the formation of well-defined F-actin rings (Jiang et al., 2015); therefore, we observed immunofluorescence of F-actin after Hes treatment. As shown in **Figure 1D**, RANKL stimulation generated the characteristically polarized F-actin ring of BMMs, while treatment with Hes remarkably diminished the number and size of F-actin rings, signifying potentially impaired osteoclast function. This was further verified by SEM observations in terms of osteolysis lesions present on bone slices (**Figure 1E**), demonstrating that, despite the establishment of large and deep lesions on control slices (only RANKL stimulation), Hes intervention decreased the number of resorption pits. Collectively, these results showed that non-toxic Hes effectively suppressed osteoclast formation and functionality, encouraging us to further explore the underlying anti-osteoclastogenesis effects of Hes.

### Suppressive Expression of Osteoclast Specific Genes by Non-toxic Hes *in vitro*

**FIGURE 2:**
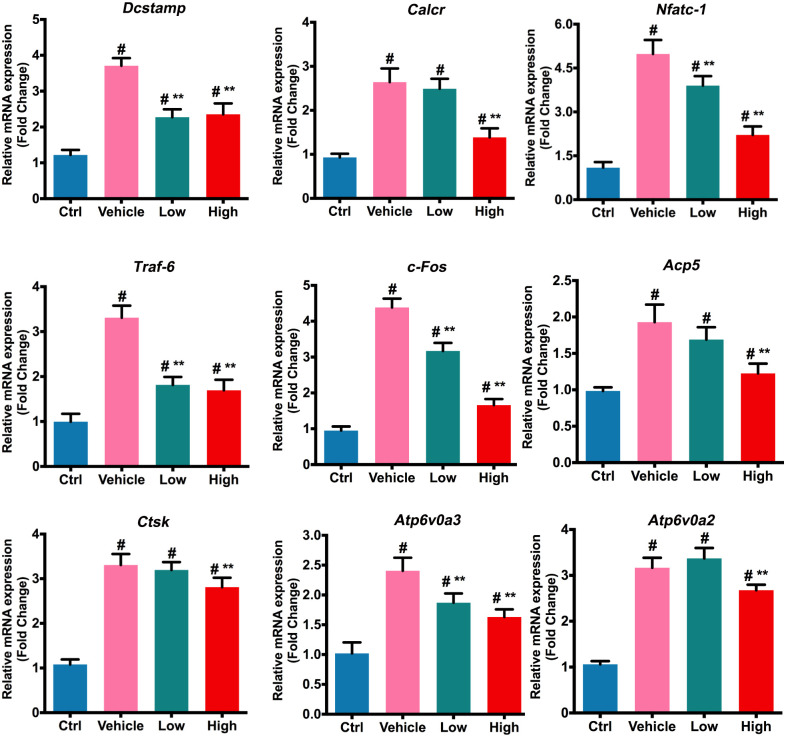
Hes mitigated RANKL-induced expression of osteoclast-specific genes. Assessment of osteoclastic effector gene expression after RANKL and Hes (low: 15 μM, high: 30 μM) treatments on mRNA levels. ^#^*p* < 0.05 compared with controls, ^∗∗^*p* < 0.05 compared with RANKL-treated vehicle.

Upon stimulation of RANKL in osteoclast precursors, a number of osteoclast-specific genes were activated, leading to the subsequent differentiation toward multinucleated osteoclasts (Boyle et al., 2003). As shown in **Figure 2**, expression of these genes, such as *Dcstamp*, *Calcr*, *Nfatc-1*, *Traf-6*, *c-Fos*, *Acp5*, *Ctsk*, *Atp6v0a3*, and *Atp6v0a2*, was induced following RANKL stimulation. However, both low and high concentrations of Hes attenuated the activation of *Dcstamp*, *Nfatc-1*, *Traf-6*, *c-Fos*, and *Atp6v0a3* markedly in comparison with vehicle (RANKL-treated only), indicating significant inhibition by Hes in terms of osteoclast gene expression levels. Additionally, although the low dosage of Hes barely affected the expression of *Calcr*, *Acp5*, *Ctsk*, and *Atp6v0a2*, Hes at high concentration could still significantly down-regulate such RANKL-induced transcription compared with vehicle, indicating that the treatment with non-toxic Hes repressed the RANKL-induced expression of osteoclast-specific genes *in vitro*.

### Inhibition of RANKL Induced NF-κB/Irf-3/c-jun/NFATc-1 Activation by Hes

**FIGURE 3:**
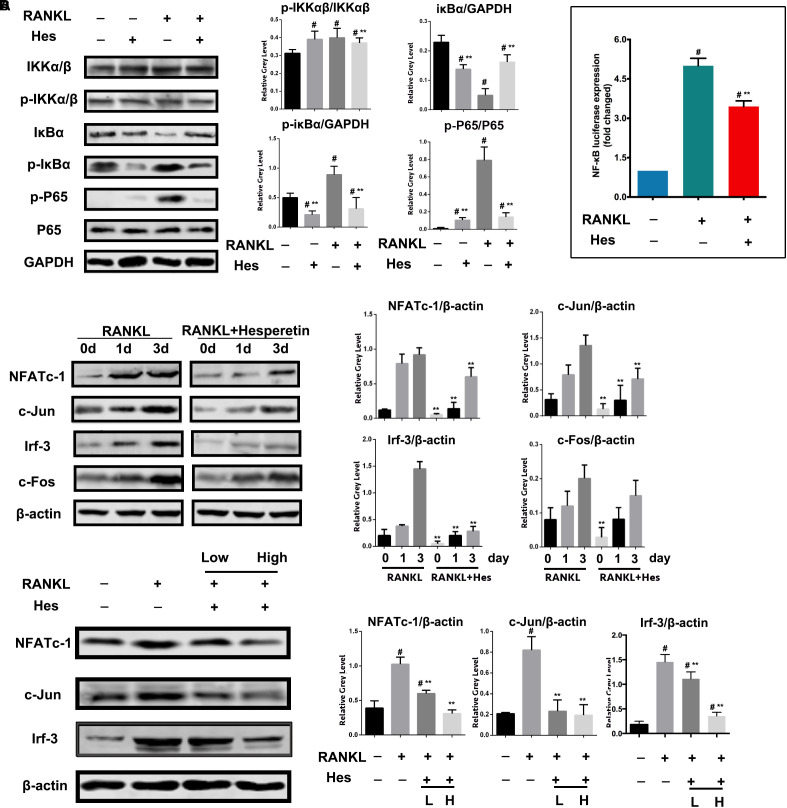
Hes inhibited RANKL-activated expressions of NF-κB signaling and downstream molecules c-Jun, Irf-3 and NFATc-1. **(A)** Evaluation of NF-κB signaling after RANKL and Hes treatments. **(B)** Evaluation of NF-κB luciferase activity after RANKL and Hes treatments. **(C)** Evaluation of NFATc-1, c-Jun, c-Fos, and Irf-3 after 1–3 days of RANKL exposure and Hes treatments. **(D)** Evaluation of NFATc-1, c-Jun, and Irf-3 after RANKL and low/high Hes treatments. ^#^*p* < 0.05 compared with controls, ^∗∗^*p* < 0.05 compared with RANKL-treated only.

It has been reported that the RANKL-induced NF-κB plays a vital role in regulating osteoclast formation. Therefore, to reveal the underlying mechanism of anti-osteoclastogenesis by Hes, we first examined the effects on NF-κB signaling by Hes treatments. **Figure 3A** shows that Hes administration tended to reduce the RANKL-stimulated expression of p-IKKα/β in comparison with RANKL-treated only. More importantly, RANKL treatment significantly facilitated the phosphorylation of p-IκBα, resulting in the effective degradation of IκBα. However, this was attenuated by treatments with Hes, as demonstrated by the decreased level of p-IκBα, plus the inhibited degradation of IκBα. In addition, despite the significant activation of p-P65 compared with control after RANKL treatment, Hes was able to suppress the promotion of p-P65. In addition, luciferase assays demonstrated that Hes repressed the RANKL elevated activity of NF-κB (**Figure 3B**). These results indicated that Hes could significantly inhibit RANKL-stimulated NF-κB activation during osteoclast formation and differentiation.

Furthermore, during the process of osteoclast formation, we found that Hes inhibited the expression of NFATc-1 after 1–3 days of RANKL stimulation. In addition, c-Jun was significantly decreased after Hes treatment compared with RANKL-treated only, whereas c-Fos was barely affected (**Figure 3C**). Notably, previous studies suggested that Irf-3 is intimately correlated with the transcriptional activity of NF-κB (Sun et al., 2014). Here, we identified that Hes administration significantly contributed to the downregulation of Irf-3 levels in osteoclasts during 3 days of RANKL treatment. After RANKL administration over 7 days, both high and low concentrations of Hes led to the decreased expression of NFATc-1, c-Jun and Irf-3 compared with the RANKL-stimulated group (**Figure 3D**), indicating that Hes could modulate NF-κB/Irf-3/c-Jun-mediated NFATc-1 activation, thereby suppressing RANKL-induced osteoclast formation.

### Inhibition of Jnk Mediated Irf-3/c-Jun Activation by Hes

**FIGURE 4:**
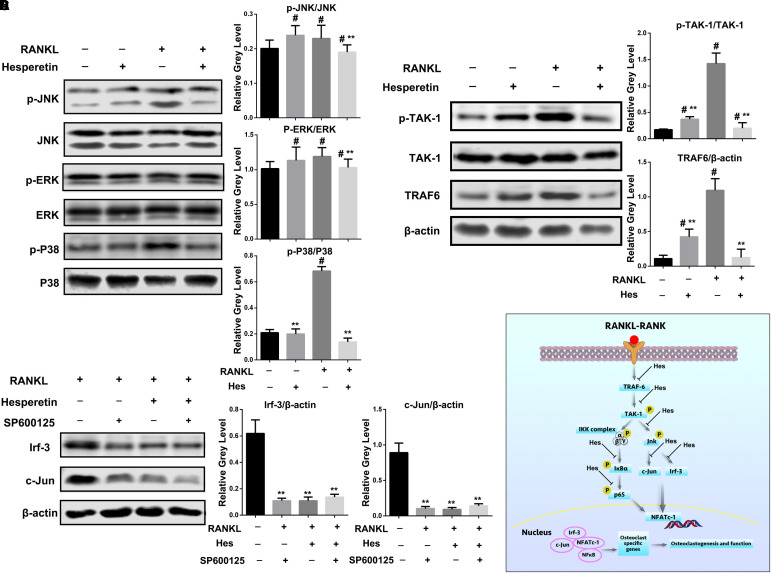
Hes inhibited Jnk-mediated Irf-3/c-Jun activation. **(A)** Evaluation of MAPK signaling after RANKL and Hes treatments. ^#^*p* < 0.05 compared with controls, ^∗∗^*p* < 0.05 compared with RANKL-treated only. **(B)** Evaluation of upstream molecules p-TAK-1/TAK-1 and TRAF-6 after RANKL and Hes treatments. ^#^*p* < 0.05 compared with controls, ^∗∗^*p* < 0.05 compared with RANKL-treated only. **(C)** Evaluation of Irf-3 and c-Jun after RANKL, Hes, and selective Jnk inhibitor treatments. ^∗∗^*p* < 0.05 compared with controls. **(D)** Schematic diagram of the possible mechanism of Hes-mediated inhibition of Jnk-mediated Irf-3/c-Jun activation.

We further explored the effects on MAPK signaling by Hes treatments. **Figure 4A** shows that in addition to the increased expression of p-Jnk after RANKL stimulation, Hes could significantly inhibit the phosphorylation of Jnk. In addition, the production of phosphorylated p38 was increased after RANKL administration, while Hes was able to diminish such a tendency. However, no obvious decrease was witnessed in the Hes-treated group in terms of p-Erk expression, indicating that p-Erk was not highly involved in the regulatory effects against osteoclastogenesis by Hes.

Furthermore, since Jnk was reported to modulate the function of Irf-3 (Zhang et al., 2009), we sought to investigate the upstream molecules regulating the RANKL-induced activation of Jnk/Irf-3/c-Jun. It was demonstrated that RANK-RANKL binding induced up-regulation of TRAF-6 and TAK-1 phosphorylation to modulate the activation of NF-κB and MAPK (Boyle et al., 2003; Singh et al., 2012). Therefore, the expression of TRAF-6 and p-TAK-1 were assessed (**Figure 4B**). We found that compared with RANKL-treated only, Hes administration could significantly inhibit the expression of TRAF-6 and p-TAK-1, indicating that Hes was capable of targeting upstream TRAF-6 and p-TAK-1 to affect downstream Jnk/Irf-3/c-jun.

In addition, SP600125, a selective Jnk inhibitor, was administered to osteoclasts for investigating the regulatory effect of Jnk on Irf-3 and c-Jun. As shown in **Figure 4C**, RANKL stimulation could elevate the expression of Irf-3 and c-Jun, which were downregulated by the treatment with SP600125, showing possible effects of Jnk in regulating Irf-3 and c-Jun. Similarly, Hes was able to decrease Irf-3 and c-Jun, implicating the possible similarity between Hes and SP600125 in inhibiting Jnk activity for down-regulating Irf-3 and c-Jun. Interestingly, after co-administration of Hes and SP600125, the levels of Irf-3 and c-Jun were decreased significantly, indicating that Hes could target Jnk for inhibiting downstream Irf-3 and c-Jun production. Collectively, by targeting the RANK-RANKL axis during osteoclastogenesis, Hes could inhibit the expression of Jnk-mediated Irf-3/c-Jun activation, thereby attenuating osteoclast formation and functionality (**Figure 4D**).

### Osteogenic Differentiation by Hes *in vitro*

**FIGURE 5:**
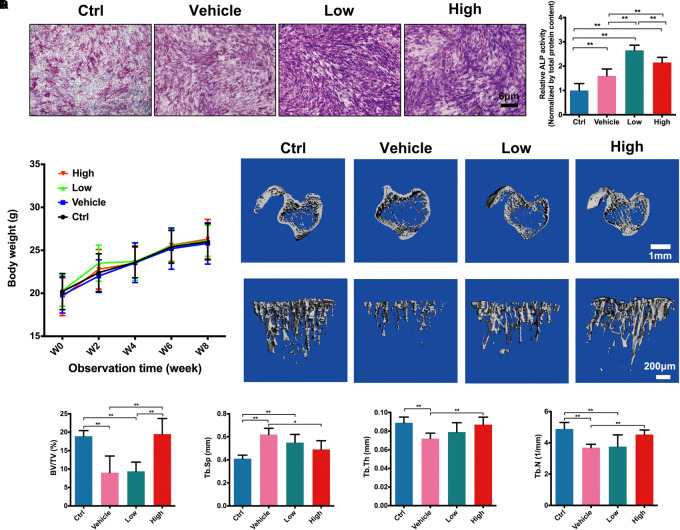
Hes promoted osteoblast formation and inhibited OVX-induced bone loss *in vivo*. **(A)** ALP staining and activity of osteoblasts after osteogenic medium and Hes treatments. **(B)** The change of body weight of experimental animals. **(C)** μCT images of tibias in OVX mice after Hes administration. **(D)** Quantitative analyses from μCT scanning revealing BV/TV, Tb.Sp, Tb.Th, and Tb.N. ^∗∗^*p* < 0.05.

As shown in **Figure 5A**, both ALP staining and ALP activity (normalized by the total protein level) of MC3T3-E1 was significantly enhanced after treatment of osteogenic medium in vehicle group. Furthermore, low and high concentrations of Hes could elevate the staining and activity of ALP significantly in comparison with vehicle. Interestingly, the osteogenic effect of low Hes was more efficient than that of high Hes, indicating that in addition to the significant anti-osteoclastogenesis effects of low Hes, it could also promote osteoblast differentiation effectively *in vitro*.

### Inhibition of OVX-Induced Bone Loss by Hes *in vivo*

After the evaluation of anti-osteoclastogenesis *in vitro*, we further assessed the inhibitory effects of Hes in regulating OVX-induced bone loss *in vivo*. We performed animal experiments of four dosages of Hes in vivo, namely 3, 6, 12, and 24 mg/kg for preliminary experiment. Results showed that 3 mg/kg Hes failed to show significant anti-osteoclastogenesis effects and 24 mg/kg Hes seemed to inhibit the increase of body weight slightly (data not shown). Therefore, 6 and 12 mg/kg Hes were selected for further experiment. As shown in **Figure 5B**, all the experimental animals showed a stable increase of body weight during 8 weeks observation, demonstrating that Hes treatments did not contribute to any obvious toxicity for normal cells. In **Figure 5C**, μCT scanning showed that OVX mice (vehicle group) developed a significant osteoporosis phenotype in tibiae trabecular structures compared with non-operated mice (ctrl group). However, after administrations of Hes intraperitoneally, both low and high concentrations of Hes could preserve the physiologic architecture of trabecular bone, as exemplified by increased density and better bone structure. These findings were further verified by the data obtained from bone histomorphometric observations (**Figure 5D**), showing that treatments with Hes were able to increase BV/TV, Tb.Th and Tb.N, whereas Tb.Sp was decreased in OVX mice. These results showed that in addition to the well-evidenced *in vitro* results showing effective anti-osteoclastogenesis by Hes, *in vivo* results implied that Hes could also demonstrate considerable efficacy in rescuing bone loss *in vivo*.

### Inhibition of OVX-Induced Osteoclast Activation by Hes *in vivo*

**FIGURE 6:**
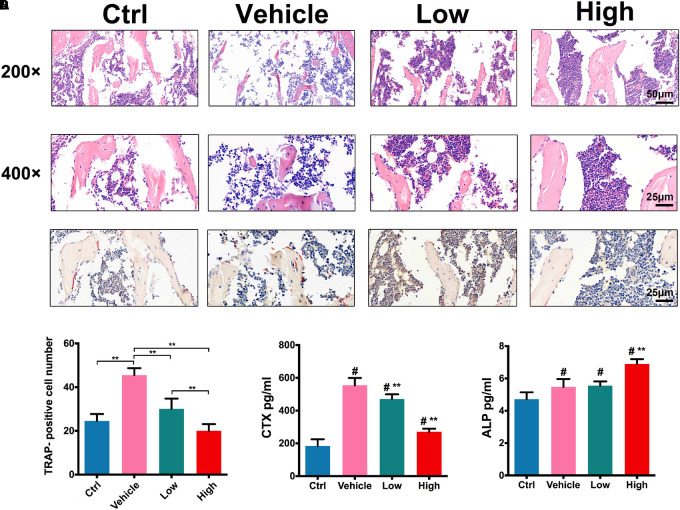
Hes attenuated OVX-induced osteoclast activation *in vivo*. **(A)** H&E staining of tibias in OVX mice administered Hes (magnification: 200×/400 ×). **(B)** TRAP staining of tibias in OVX mice administered Hes (magnification: 400×). **(C)** Quantitative analyses of TRAP-positive osteoclasts in tibias of OVX mice administered Hes. ^∗∗^*p* < 0.05. **(D)** Serum levels of osteoclastic CTX and osteoblastic ALP from OVX mice administered Hes. ^#^*p* < 0.05 compared with ctrl-treated animals, ^∗∗^*p* < 0.05 compared with vehicle.

H&E staining assessment (**Figure 6A**) showed that, in comparison with the ctrl group, OVX induced significant bone loss in trabecular bone in vehicle group, showing scarce bone volume with impaired trabecular structures. However, the treatments with Hes gradually increased the bone volume of trabecular in a dose-dependent manner, exhibiting increased trabecular density and the preservation of bone structure. **Figures 6B,C** further show the staining of TRAP-positive osteoclasts *in vivo*. Results indicated that OVX surgery significantly increased the number of TRAP-positive osteoclasts, while Hes treatments alleviated OVX-induced osteolytic lesions, with decreased numbers of TRAP-stained, mature osteoclasts in trabecular bone, implying the effective anti-osteoclastogenesis effects of Hes *in vivo*.

In addition, CTX that acts as a bone resorption index and ALP that serves as an osteoblastic marker were deployed to evaluate changes in serum indicators. As shown in **Figure 6D**, OVX induced the significant elevation of CTX levels compared with ctrl-operated mice. Nonetheless, treatments with Hes dose-dependently reduced such a tendency, as evidenced by the gradual decrease of CTX levels in Hes-treated mice. In addition, the vehicle-only mice developed a slight increase in ALP production compared with ctrl animals, possibly due to the reason that activated osteoclasts, in turn, facilitated reciprocal osteoblastogenesis. Furthermore, Hes treatments increased the production of ALP in OVX mice, which was consistent with previous results (Xue et al., 2017), indicating the potent possibility for future translational practice involving Hes to attenuate osteoclastogenesis and promote osteoblastogenesis.

Taken together, these results demonstrated that Hes alleviated RANKL-induced osteoclast activation and function *in vitro*, and attenuated OVX-induced osteoporosis *in vivo*. More importantly, for the first time, we showed the significance of Irf-3 in regulating osteoclastogenesis, providing a novel molecular target for the development of future anti-osteoporosis therapeutic strategies. It was demonstrated that by inhibiting RANKL-induced activation of Jnk, Hes was able to attenuate the downstream expression of Irf-3 and c-Jun significantly, thereby inactivating NF-κB/MAPK signaling and the transcription factor NFATc-1, leading to the suppressive levels of osteoclast specific genes which resulted in impaired osteoclastogenesis and functionality.

## Discussion

To date, much progress has been accomplished to treat osteoporosis, whereas further advances involving pharmaceutical targets and therapies are still in demand. Herein, we found that Irf-3 played a vital role in RANKL-induced osteoclastogenesis, during which Jnk mediated Irf-3/c-jun activation was down-regulated, showing effective inhibition upon osteoclast formation and function both *in vitro* and *in vivo*.

Osteoporosis features diminished bone mineral density (BMD) and increased bone fragility, and has been shown to derive from the unbalanced activities of bone resorption and formation (Xie et al., 2014; Marozik et al., 2018). Currently, bisphosphonate (e.g., alendronate, risedronate), selective estrogen receptor modulators (SERMs) and RANKL antibody (denosumab) (Maximov et al., 2013; Pazianas and Abrahamsen, 2016; Qiao and Tang, 2018) are representative components inhibiting osteoporosis. However, the accompanying inevitable side effects such as fever, hypercalcemia and hypertension, thromboembolism, atypical fractures, severe gastrointestinal disorders, and osteonecrosis of the jaw (ONJ) (Tian et al., 2014) largely restrain their clinical application, which propels the development of novel pharmaceutical remedies to affect osteoclast formation without cytotoxicity. Herein, Hes showed effective anti-metabolic influence against osteoporosis, indicating that non-toxic concentrations of Hes were able to inhibit RANKL-induced osteoclast differentiation and maturation among three types of osteoclast precursors. These observations were further confirmed by the inhibited formation of F-actin rings and resorption pits on bone slices, enabling us to further explore the underlying mechanisms for providing new molecular targets for future osteoporosis treatments.

The stimulation of RANKL to mediate the trimerization of RANK, the cell-surface receptor on preosteoclasts, promotes the activation of a series of intracellular molecular responses (Zhou et al., 2017; Ouyang et al., 2018). The activation of TRAF-6 initially induces the expression of phosphorylated TAK-1, which leads to the subsequent phosphorylation of IKKα/β, Jnk, Erk, and p38, all of which are vital for osteoclastogenesis. The classic NF-κB signaling involves activation of p-IκBα followed by increased p-IKKα/β to degrade IκBα, enabling the following phosphorylation and nuclear translocation of NF-κB protein, P65 (Abu-Amer, 2013). These biological events involving the NF-κB pathway contribute to the normal maturation and function of osteoclasts to exhibit bone resorption activity. Together with NF-κB, MAPK proteins (Jnk, Erk, and p38) increase the enhanced expression of osteoclast specific genes, which act as the functional molecules during osteoclastogenesis (Boyle et al., 2003; Singh et al., 2012; He et al., 2014). Among these osteoclast effector genes, c-Jun and NFATc-1 have been considered as the leading factors affecting osteoclastogenesis (Boyce et al., 2015). It was reported that c-Jun could induce NFATc-1, a significant regulator that reinforces osteoclast formation. This explains our findings that inhibition of NF-κB and MAPK by Hes not only led to the suppression of RANKL-stimulated c-Jun and NFATc-1, but also repressed the downstream osteoclast-specific effector genes such as *Dcstamp*, *Calcr*, *Nfatc-1*, *Traf-6*, *c-Fos*, *Acp5*, *Ctsk*, *Atp6v0a3*, and *Atp6v0a2* dose-dependently. Our results showed that by inhibiting RANKL-stimulated phosphorylation of Jnk in osteoclast precursors, Hes could mitigate the downstream levels of Irf-3 and c-Jun markedly, thus inactivating NF-κB/MAPK signaling and transcription factor NFATc-1, leading to suppressive levels of osteoclast-specific genes that resulted in impaired osteoclastogenesis and functionality. Previously, we screened five drug candidates to treat osteoclast precursors; however, only Hes showed the consequences of Irf-3 regulatory effects during RANKL-induced osteoclastogenesis (data no shown). To the best of our knowledge, this is the first detailed study covering the importance of Irf-3 during the treatment of osteoclast-related diseases, extending the possibility of applying Hes to target Irf-3 in other osteoclastic diseases such as cancer bone metastasis, osteoarthritis, bacterial infection and wear-particle-induced calvarial osteolysis.

In summary, for the first time we reported the significant regulatory effect of Irf-3 in regulating RANKL-induced osteoclastogenesis. By the application of Hes to osteoclast precursors, Jnk-mediated Irf-3/c-Jun activation was inhibited, thus attenuating RANKL-induced osteoclast formation and function, both *in vitro* and *in vivo*. However, major challenges and limitations still remain in this study. First, in addition to osteoclast differentiation, more investigation of the osteoclast proliferation and apoptosis is required, which could deepen the understanding of Hes treatments against osteoporosis. Second, the results of Hes administration on other skeletal parameters like spine/femur BV/TV, bone length, tibial growth plate height or dynamic histomorphometry/osteoblast parameters are demanded in future preclinical exploration. Third, although Irf-3 plays significant roles during osteoclastogenesis, its potential role during osteoblastogenesis after Hes treatments still requires further investigation. Overall, despite above limitations, there is significant room to improve Hes treatments for better understanding the underlying mechanisms during bone remodeling.

## Author Contributions

QZ and XT carried out the molecular genetic studies, participated in the sequence alignment, and drafted the manuscript. XT carried out the animal study. ZL carried out the immunoassays. XS participated in the sequence alignment. DP and WZ participated in the study design and performed the statistical analysis. ZO and WW conceived of the study and participated in its design and coordination, and helped to draft the manuscript. All authors read and approved the final manuscript.

## Conflict of Interest Statement

The authors declare that the research was conducted in the absence of any commercial or financial relationships that could be construed as a potential conflict of interest.
