# Correction: Hesperetin prevents bone resorption by inhibiting RANKL-induced osteoclastogenesis and Jnk mediated Irf-3/c-Jun activation

**DOI:** 10.3389/fphar.2025.1678215

**Published:** 2026-01-07

**Authors:** Qiang Zhang, Xinqiao Tang, Zhong Liu, Xiaoxia Song, Dan Peng, Wei Zhu, Zhengxiao Ouyang, Wanchun Wang

**Affiliations:** 1 Department of Orthopedics, The Second Xiangya Hospital, Central South University, Changsha, China; 2 Department of Orthopedics, Xiangtan Central Hospital, Xiangtan, China; 3 Department of Respiratory Medicine, Xiangtan Central Hospital, Xiangtan, China

**Keywords:** osteoporosis, hesperetin, RANKL, Irf-3, MAPK, NFATc-1

There was a mistake in Figure 1C (control and 30 μM groups) as published. During figure preparation, the images for the splenocytes control group and the 30 μM group in Figure 1C were incorrectly selected, resulting in a misrepresentation of the experimental data. The corrected Figure 1 appears below.

**FIGURE 1:**
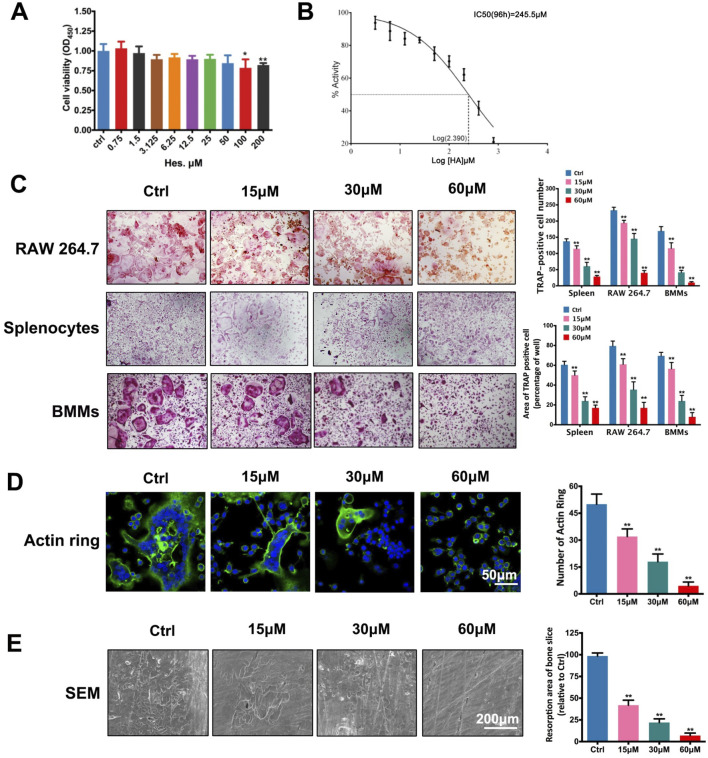
Non-toxic Hes attenuated RANKL-induced osteoclast formation and function *in vitro*. **(A)** Cell viability of osteoclast precursors after Hes treatments for 24 h. **(B)** Linear correlation between OD values and cell numbers. **(C)** RANKL-induced osteoclastogenesis after Hes treatments in three types of preosteoclasts, RAW 264.7 cells, splenocytes, and BMMs. **(D)** Formation of RANKL-induced F-actin rings after Hes treatments. **(E)** Formation of RANKL-stimulated bone resorption pits after Hes treatments. *p < 0.05 compared with controls, **p < 0.01 compared with controls.

This error does not affect the interpretation or conclusions of the article in any way.

The original article has been updated.

